# Omega‐3 Fatty Acids, Lecithin, Sterols, Vitamins and Lipid Oxidation of Olein and Super Olein Fractions of Fish Oil Produced by Winterization

**DOI:** 10.1002/fsn3.4754

**Published:** 2025-01-15

**Authors:** Aiza Khaliq, Muhammad Hafeez‐ur‐Rehman, Farzana Abbas, Muhammad Nadeem, Muhammad Abdul Rahim, Fahad Al‐Asmari, Mohamed Fawzy Ramadan, Eliasse Zongo

**Affiliations:** ^1^ Department of Fisheries & Aquaculture University of Veterinary & Animal Sciences Lahore Punjab Pakistan; ^2^ Department of Dairy Technology University of Veterinary & Animal Sciences Lahore Punjab Pakistan; ^3^ Department of Food Science & Nutrition Faculty of Medicine and Allied Health Sciences, Times Institute Multan Pakistan; ^4^ Department of Food and Nutrition Sciences, College of Agricultural and Food Sciences King Faisal University Al‐Ahsa Kingdom of Saudi Arabia; ^5^ Department of Clinical Nutrition, Faculty of Applied Medical Sciences Umm Al‐Qura University Makkah Saudi Arabia; ^6^ Laboratoire de Recherche et d'Enseignement en Santé et Biotechnologies Animales Université Nazi BONI Bobo Dioulasso Burkina Faso

**Keywords:** fish oil, lipid oxidation, olein, omega‐fatty acids, super olein, winterization

## Abstract

To concentrate omega‐3 fatty acids (*n*‐3) in fish oil (FO), olein and super olein fraction (OF) of FO were produced by winterization. For this purpose, FO was slowly cooled to −50°C (24 h), the mixture of crystallized and non‐crystallized phases was separated, filtrate was coded as OF (yield 32%), 35% of OF was kept for storage study and analytical purpose, remaining 65% was further slowly cooled down to −75°C (24 h) and filtered, filtrate was coded as super olein (SF, yield 23%). GC–MS analysis showed that unsaturated fatty acids increased due to successive winterization. In OF, C18:1, C18:2, C18:3, C20:1, C20:5 (EPA), C22:1, C22:2 and C22:6 (DHA) increased to 7.85%, 19.52%, 54.16%, 17.82%, 16.31%, 41.02%, 32.43%, and 29.89% than parent FO. In SF, C18:1, C18:2, C18:3, C20:1, C20:5, C22:1, C22:2 and C22:6 increased to 9.84%, 24.35%, 61.09%, 32.10%, 39.96%, 56.81%, 39.02%, and 48.94% than parent FO. Total phenolic contents (TPC) of FO, OF, and SF were 6.59, 12.67 and 19.72 (mgGAE/mL). Lecithin content of FO, OF, and SF were 1.29%, 0.575%, and 0.19%. In SF, desmosterol, cholesterol, stigma sterol and sitosterol were 91.57, 22.51, 12.67 and 112.18 mg/100 g. Total antioxidant capacity (TAC) of FO, OF, and SF was 49.32%, 64.27%, and 85.47%. DPPH values of FO, OF, and SF were 32.14%, 39.87%, and 46.41%. Winterization significantly raised vitamin A and E in OF and SF; vitamin A content in FO, OF, and SF (0‐day) were 46.28, 67.94, and 116.48 IU; vitamin E content in FO, OF, and SF (0‐day) were 1238.95, 1897.65, and 2375.11 mg/100 g. At 0‐day, peroxide value (POV) of FO, OF and SF was 0.22, 0.24 and 0.25 (MeqO_2_/Kg) with no variation in sensory characteristics. The results of this study proved that *n*‐3 could be increased in olein and super OFs of FO with reasonable oxidative stability.

## Graphical abstract

In the present investigation, the super olein fraction (OF) of fish oil (FO) was first time produced by winterization. EPA and DHA contents of super OFs were 39.7% and 48.9% higher than parent FO, respectively. In the OF, EPA and DHA were 16.3% and 29.8% more than substrate FO, respectively. After 90 days of ambient storage, the sensory properties of free fatty acids, peroxide value, and super OFs were similar to those of fish oil. Omega‐3 (*n‐3*) fatty acids can be successfully increased in the super OF of FO by winterization.

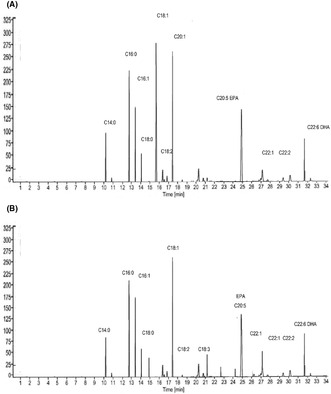

## 1 Introduction

Omega‐3 (*n‐3*) are polyunsaturated fatty acids having a double bond on the third carbon of the chain of hydrocarbons. Beneficial impacts of (*n*‐3) include reducing the risk of heart diseases, developing the brain, retina, and heart, and preventing tumors, inflammation, diabetes, obesity and several lifestyle‐related disorders (Sioen et al. 2006). Eicosapentaenoic acid (EPA) and docosahexaenoic acid (DHA) are not present in staple foods of several countries. These essential fatty acids are only present in fatty fish and seafood. Shortage of *n*‐3 has been connected with cardiovascular issues, skin, mood, behavior, respiratory and diabetes issues (Lands 2005). In a healthy diet, two servings per week of fatty fish should be present to obtain the essential *n*‐3 in the desired amount. However, fish is not consumed in several countries of the word due to its limited supply in winter months or due to several financial, cultural, availability, and sustainability issues. Communities taking inadequate fish cannot get beneficial vitamins A, E, *n*‐3 and numerous bioactive compounds. In such conditions, intake of fish oil (FO) or the supplementation of foods with FO should be performed to transport *n*‐3 fatty acids to the body (Ribarova 2007). The average content of EPA and DHA in FO is approximately 12%–15%. For the fortification of foods to carry *n*‐3 to the body in the desired amount (2 g/day), a considerable amount of FO is needed (Health Canada 2008). Further, the FO used for food fortification will also carry a substantial amount of unnecessary oil in the food matrix. The use of FO in the production of fortified foods for a longer period may leads to fatty liver, diabetes type 2 and hyperlipidemia thus disturbs energy imbalance and leads to organ damage (liver, kidney, and heart), moreover the current strategy of food fortification with fish oil also leads to the wastage of prestigious source of *n*‐3; therefore, innovation in the substrate FO for the fortification purpose is needed for the ever‐increasing issue of food insecurity and metabolic disorders (Breivik 2007). In addition to *n*‐3, FO is a good source of lecithin, a multi‐blend of components of which phospholipids are most common, whereas a minor quantity of sterols, tocopherols, fatty acids and glycolipids are also present. It is a most common emulsifier, widely used in food industries, and commercially extracted from soya beans through the degumming process (Nieuwenhuyzen and Tomas 2008; Topuz et al. 2021) *n*‐3 enriched FO may be used to develop sustainable food fortification strategies. Several methods of increasing unsaturated fatty acids in edible oils have been discovered, such as solvent fractionation, urea encapsulation, dry crystallization/winterization, etc. Due to the potential toxicity of solvents and lower yield in the first two methods, it may not be safer for the consumer and economically viable to increase *n*‐3 in the substrate FO by winterization (Van Aken et al. 1999). Winterization, the green modification process, is an industrially adopted method, and a substantial amount of vegetable oils like palm oil is fractionated to produce OF, mid‐fraction and SF. Two or more fractionations are separated in this method, from oils based on differences in melting point to remove disaturated and trisaturated glycerides Sunflower oil is fractionated/dewaxed by this method (Shahidi 2005). In a recent investigation, date seed oil was fractionated by winterization procedure to produce OF and SF fractions, oleic acid content of OF and SF was considerably higher than parent date seed oil. (Hussain et al. 2022) By winterization, OF, SF, and mid‐fraction of FO can be produced, which can be combine together with other vegetable oils such as, soybean and sunflower oils for the fortification food products, chocolates, value‐added bakery, and dairy products. The substantial amount of vegetable oils like sunflower oil is fractionated to produce OF, mid‐fraction and SF (Shahidi 2005). The advancement in technology has increased comfort levels in daily lives, but it has shifted the disease pattern as well; with a gap of one generation, people had more physical activity, and they were used to having more infectious diseases, but in existing lifestyles, challenges of metabolic ailments have surpassed the infectious diseases. Developing functional foods with bioactive compounds using technological methods is further recommended. To increase *n*‐3, flaxseed oil was cooled to −40°C, and −60°C to obtain OF and SF fractions; *n*‐3 in flaxseed oil, olein and SF fractions were 55, 61% and 71%, respectively (Azad et al. 2021). Compared to saturated fatty acids, *n*‐3 is more easily oxidized; food fortification strategies focused on developing *n*‐3 enriched foods can only be successful and sustainable if lipid oxidation is controlled at all stages of food processing, packaging and distribution (Nadeem et al. 2017). Nutritionists believe consuming oxidized dietary lipids may be more dangerous for the health (Ullah et al. 2016). *n* *‐3* fatty acids are polyunsaturated fatty acids that are easily prone to lipid oxidation, thus oxidized form of *n* *‐* *3* lose their therapeutic qualities and leads to production of toxic oxidative and off‐flavor products like alcohols, ketones, and aldehydes (Goyal et al. 2014). Research findings have revealed that oxidation products increase the risk of atherosclerosis and carcinogenesis (Hussain et al. 2022). Oxidation of *n*‐3 can lead to the development of painty, grassy and other highly undesirable flavors that can lead to sensory and quality defects in food products (Nadeem and Imran 2016). FO has a reasonable amount of *n*‐3 that may be included in functional foods; however, FO with a higher magnitude of *n*‐3 will deliver more *n*‐3 with a minimum amount of unnecessary oil. Therefore, studying the oxidative stability of OF and SF in FO is extremely important. A winterized version of FO has already been produced; however, SF from FO is the need of the hour. Fatty acid profile, lipid oxidation, sterols and vitamin content of olein and SF of FO should be comprehensively studied for probable application in foods. Fractions of FO containing higher amounts of *n‐* *3* fatty acids may be used for the production of baby foods, infant formulae, margarines, chocolates, yoghurt, and several food products. Therefore, this study aims to determine the impact of winterization on fatty acid profile, lipid oxidation, lecithin, sterols, sensory properties and vitamin content of both OF and SF of FO through advanced analytical techniques.

## 2 Materials and Methods

### 2.1 Production of OF and SF

Fish oil (FO) was slowly cooled to −50°C (24 h), the mixture of crystallized and non‐crystallized phases were separated on a Buchner funnel attached with a vacuum pump at a pressure of −400 mmHg, filtrate was coded as olein fraction (OF, yield 32%), 35% of OF was kept for storage study and analytical purpose, remaining 65% was further slowly cooled down to −75°C (24 h), crystallized and non‐crystallized phases were again separated in the same way as stated above, filtrate was coded as super olein (SF, yield 23%). To minimize the variation, each fractionation processes was repeated at least six times and each was regarded as a replicate. FO, OF and SF were filled in transparent glass bottles, stored between 22°C–26°C sampled at 0, 45 and 90 days for sensory and chemical analysis (Khan et al. 2018).

### 2.2 Chemical Analysis of FO, OF, and SF

FO, olein and super olein fractions of FO were analyzed for free fatty acids (FFAs), saponification value, unsaponifiable matter, refractive index, iodine value (IV), peroxide value (PV) and color was determined by following the standard methods (AOCS 2011).

### 2.3 Fatty Acid Composition

Fatty acid determination was done on GC–MS (7890‐B, Agilent Technologies) using column SP‐2560 (100 m × 0.25 mm id: 0.20 μm) equipped with Flame Ionization Detector (FID). Briefly, methyl esters were prepared, and a 50 g sample was reacted with 2 mL (HCL) in 15% C_2_H_5_OH on a hotplate for 1 h at 100°C. A stay time of 15 min was given until the temperature of the sample decreased to 20°C–25°C. Further deionized water and 2 mL of 99% *n*‐hexane were added to the solution, followed by Vortex at 1500 rpm for 1 min. For injection, the test tubes upper layers were removed, and 1 μL sample and remaining dried Na_2_SO_4_ were added in GC vials at a split ratio of 1:50. The temperature of FID, injector and oven was set to 260°C, 250°C and 225°C, respectively. He, O_2_ and H were flowing at 2, 4 and 40 mL/min. For identification and quantification, FAME‐37 was used as standard (Qian 2003).

### 2.4 Total Phenolic Contents (TPC)

TPC in the oil sample were determined in the freshly extracted FO, olein and super olein fractions using the Ciocalteu method with minor modifications. Briefly, 200 μL of oil sample was mixed with freshly prepared Folin–Ciocalteu reagent, diluted with 20%, w/v (sodium carbonate) and 1:2, v/v (water) and mixed with gallic acid (taken as calibration standard of various strengths; *R* ^2^ = 0.9916) the concentration of TPC was determined from the calibration curve by using the following expression (Terpinc et al. 2012).

$$A=0.98C+9.925\times 10^{-3}$$

### 2.5 Determination of Lecithin

FO was dissolved in 95% of 100 mL ethanol and stirred for 6 h by a magnetic stirrer. The mixture was centrifuged for 10 min at 1900 rpm and filtered through filter paper. The supernatant was collected in a beaker and residue was again subjected to centrifugation for further extraction of neutral and polar lipids. Collected supernatants were mixed (200 mL) and 400 mL of hexane was added to obtain neutral lipids. For extraction of neutral lipids from polar lipids, 200 mL of hexane was added to the former 100 mL ethanol extract. In a rotary evaporator (WEV‐1001 L) ethanol extract was evaporated at 40°C. The residue left was dissolved in hexane. The fifth part of chilled acetone (−4°C) was added to the hexane mixture and stirred until the precipitation of viscous lecithin. For 15 min this mixture was placed in an ice bath and centrifuge at 1500 rpm for 10 min. The supernatant was discarded and a sticky gummy material (lecithin) was stored at −40°C for further analysis. Extracted lecithin was determined on GC–MS (Agilent Technologies) using column (100 m × 0.25 mm id: 0.20 μm) equipped with Flame Ionization Detector (FID). The extracted sample (2.0 mL) was injected in the GC–MS, and the determination of fatty acid methyl of the extracted sample was done by comparing the retention time of peaks with the standard sample. The detector temperature was set to 250°C, whereas the inlet temperature was set to 200°C. Hydrogen, Helium and oxygen flow rate was set to 4 mL/min, 2 mL/min and 40 mL/min (Abiona et al. 2021).

### 2.6 Sterols

Samples of FO 0.3 g were taken in test tubes in which 3 mL ethanolic solution of NaOH (2 M) and 10 μL, 5α‐cholestase (0.5 mg/mL) were added and vortex for 1 min at 1500 rpm. The prepared solution was heated in a water bath for 15 min at 90°C. Further *n*‐hexane and 2 mL of deionized water were added, and the solution was again centrifuged at 1500 rpm for 10 min. Under N_2_, the hexane was evaporated, and the remaining sample was further treated with 10 μL of Tri‐Sil reagent for 30 min. Hexane was added to the sample, and the prepared solution was transferred to GC vials for automatic injection by an auto liquid sampler of GC–MS (7890‐B, Agilent Technologies) with fused silica capillary column DB‐5HP (30 m × 0.32 × 0.1 μm). The temperature of the flame ionization Detector was set to 260°C, and oven temperature was increased from 50°C to 315°C at 40°C/min. Helium, Hydrogen and Oxygen were circulated in the system at 2, 4 and 40 mL/min. Sterols were identified by internal standards (Azadmard‐Damirchi 2010).

### 2.7 Total Antioxidant Capacity (TAC)

Sample was taken (100 μL) and mixed in reducing species (hexane, methanol, dimethyl sulfoxide and water) and 1 mL reagent solution in test tube was added. Reagent solution was prepared by mixing 28‐Mm sodium phosphate, sulfuric acid 0.60‐M and 4‐mM ammonium molybdate. Oil sample was heated in heating block at 95°C further it was cooled to room temperature (25°C–30°C). TAC was determined on a double‐beam spectrophotometer, absorbance was recorded at 695 nm, and values were expressed in percentage. Total antioxidant property was denoted as 5 standards of ascorbic acid, and α‐tocopherol (Al‐Farsi et al. 2005).

### 2.8 1,1‐Diphenyl‐2‐Picrylhydrazyl (DPPH)

DPPH solution (20 mg/L) was prepared in C_2_H_5_OH. Briefly, 1.5 mL DPPH solution was mixed with an oil sample (70 μL). The tubes were kept in dark conditions for a time duration of 30 min. Absorbance was recorded on a double‐beam spectrophotometer at 517 nm, and values were expressed in percentage (Al‐Farsi et al. 2005).

### 2.9 Analysis of Vitamin A and E by HPLC

Fat‐soluble vitamins, namely Vit A and Vit E, were analyzed using reverse phase HPLC equipped with a controller, a UV‐DAAD fluorescence detector, and a C18 controller (1260 Infinity^o^C, Agilent Technologies) equipped with analytical column ODS2 Hypersil 250 × 4, 6 mm, 5 μ. The mobile phase was set 97:3 = MeOH: H_2_O at a flow rate of 1 mL/1 min. The qualitative analysis was performed by comparing the retention times of standard samples of Vit A (retinol) and Vit E (tocopherol) at λ_em_ = 288 nm and λ_em_ = 332 nm, respectively. Further, the chromatographic peaks were compared with standard samples of A (retinol) and Vit E (tocopherol) for quantification analysis. The results were expressed as μg/100 (wet weight). All the chemicals used for determination are of analytical grade and purchased from Sigma Aldrich, USA (Khan et al. 2018).

### 2.10 Lipid Oxidation

Cholesterol and lipid oxidation of FO, OF and SF was determined using POV, free fatty acid and anisidine value (AV) by standard procedures (AOCS 2011).

### 2.11 Sensory Evaluation

Samples of FO, OF, and SF were randomly coded and arranged, and color and smell in all samples were evaluated using 10 trained judges (age ranges from 30 to 43 years) who had previous experience in sensory evaluation of edible oils and fats. Before the sensory evaluation, training sessions were conducted, vocabulary was standardized. Evaluations for color and smell were performed in individual sensory evaluation booths at 20°C–25°C, distilled water was provided and data were recorded using FIZZ software (Larmond 1977).

### 2.12 Statistical Analysis

The experiment was conducted in CRD with triplication of every treatment to determine the effect of the fractionation and storage phases by one‐way and two‐way ANOVA. Normality and homogeneity of variance were verified prior to analysis. The Duncan Multiple Range Test was applied in SAS 9.4 to determine the significant variation among the means using a *p* < 0.05.

## 3 Results and Discussion

### 3.1 Chemical Characteristics of FO, OF, and SF

In winterization, two or more fractionations are separated from oils based on differences in melting point to remove disaturated and trisaturated glycerides (Van Aken et al. 1999). By winterization, OF, SF, and mid‐fraction of fish oil (FO) can be produced, which can be mixed with other vegetable oils such as canola, soybean and sunflower oils for the production of salad dressings, fortification of margarine, butter, chocolates, value‐added bakery and dairy products. Substantial amount of vegetable oils like sun oil is fractionated to produce OF, mid‐fraction and SF (Shahidi 2005). With increasing demand for *n*‐3 fatty acids and *n*‐3 enriched foods, sustainable production methods of *n‐3* supplements should be developed. Extracting *n*‐3 from FO by winterization is a physical process without involving any undesirable chemical reaction in the substrate oil. Hence, the remaining oil can be edible (Nadeem et al. 2014). Other methods of fat modification, such as interesterification either by 1–3 specific lipases or by sodium methoxide, led to the loss of about 5%–10% neutral oil. Hydrogenated oils need post‐refining to remove residues of nickel catalysts, decreasing FFA and removing the typical flavor of hydrogenation (Nadeem et al. 2017). In this study, FO was winterized twice to produce OF and SF; fractions and parent FO had similar FFA content (Table 1). FFA of FO, OF and SF were 0.13%, 0.12%, and 0.14% (oleic acid). OF and SF were developed for the probable fortification of foods with *n*‐3; lower FFA content in these fractions is essential for oxidative stability and sensory properties of fortified foods. Excessive FFA can induce off‐flavors in foods and accelerate the free radical mechanism, significantly decreasing the shelf life and sensory quality of foods (Frega, Mozzon, and Lercker 1999). No post‐winterization refining is required to lower the FFA content as these are critically monitored by regulatory bodies across the globe; the EU has allowed 0.2% (max) in foods. OF and SF may become a good source of *n‐3* supplements for food fortification and pharmaceutical applications. Non‐significant impact of winterization was recorded on other parameters such as SV, moisture and color; however, winterization induced major changes in UM, IV and RI of OF and SF, and concentrations of UM increased in OF and SF (*p* < 0.05). The unsaponifiable matter comprises fat‐soluble vitamins, phenolic, bioactive and coloring compounds. A rise in UM means OF, and SF have better functional values than parent FO. Justification for the intensification of fat‐soluble vitamins, phenolic, bioactive and coloring compounds in OF and SF by winterization was due to the intensification of low‐melting glycerides as disaturated and trisaturated glycerides were removed by the two‐stage winterization involved in the production of OF and SF (Nadeem et al. 2014). The winterized flaxseed oil had a higher extent of UM than the parent flaxseed oil (Azad et al. 2021). IV of FO, OF, and SF were 207.43, 229.53 and 254.67 cg/100 (*p* < 0.05); the rise in IV and RI of OF and SF were due to the incremental increase and reduction of unsaturated and saturated fatty acids in OF and SF (Ullah et al. 2016) produced olein and stearin fraction from chia using winterization, olein fraction had more UM, RI and IV than parent chia oil. Winterization did not affect the color of sunflower oil; raw and winterized sunflower oil had almost the same color (Kreps, Vrbikova, and Schmidt 2014). Date seed oil was fractionated by winterization procedure to produce OF and SF fractions, oleic acid content of OF and SF was considerably higher than parent date seed oil (Hussain et al. 2022).

**TABLE 1:** Chemical characteristics of FO, OF and SF.

Parameter	FO	OF	SF
FFA %	0.13 ± 0.02^A^	0.12 ± 0.01^A^	0.14 ± 0.01^A^
SV (mg KOH/g oil)	194.89 ± 0.31^A^	195.13 ± 0.47^A^	194.89 ± 0.55^A^
UM %	1.77 ± 0.03^C^	1.92 ± 0.04^B^	2.16 ± 0.02^A^
IV (Cg/100 g oil)	207.43 ± 0.17^C^	229.53 ± 0.34^B^	254.67 ± 0.61^A^
RI (@40°C)	1.4788 ± 0.01^C^	1.4793 ± 0.02^B^	1.4799 ± 0.01^A^
Moisture %	0.17 ± 0.02^A^	0.16 ± 0.01^A^	0.20 ± 0.02^A^
Color red + yellow (Lovibond scale)	20 ± 0.21^A^	21 ± 0.14^A^	22 ± 0.11^A^

*Note:* In rows of individual parameters, when means bear different letter, it means these are statistically different (*p* < 0.05).

### 3.2 Fatty Acid Profile

In the present investigation, FO was first cooled to −50°C to produce OF and then OF was further cooled to −75°C; the portion of FO solidified at each temperature was separated from the liquid portion by filtration. Solidified portions were mainly comprised of fatty acids that crystallized at the given temperature; thus, concentrations of unsaturated fatty acids increased as the winterization proceeded, leading to a significant modification in fatty acid profiles (Table 2). In winterization, solidification/ crystallization of fatty acids was dependent upon their melting points, for example, melting points of C14:0, C16:0, C18:0, C18:1, C18:2 was 44.5°C, −0.1°C, 63.2°C, 69.8°C, 13.5°C, −4.5°C, and −13.5°C, respectively (Reddy et al. 2010). The difference in the melting point of fatty acids was the reason for the significant variation in the fatty acid profile of OF and SF from FO. GC–MS analysis of FO showed the concentrations of C14:0, C16:0, C16:1, C18:0, C18:1, C18:2, C18:3, C20:1, C20:5 (EPA), C22:1, C22:2 and C22:6 (DHA) were 4.91, 13.22, 9.43, 2.63, 25.72, 2.64, 1.21, 13.88, 7.75, 0.92, 0.25 and 8.16 mg/100 g, respectively. As a result of winterization, C14:0, C16:0, C16:1 and C18:0 were decreased to 35.65%, 36.15%, 23.76%, and 26.72% in OF than parent FO. In SF, C14:0, C16:0, C16:1, and C18:0 were decreased to 76.58%, 75.27%, 59.39%, and 71.49% compared to parent FO. In OF, C18:1, C18:2, C18:3, C20:1, C20:5 (EPA), C22:1, C22:2 and C22:6 (DHA) increased to 7.85%, 19.52%, 54.16%, 17.82%, 16.31%, 41.02%, 32.43%, and 29.89% than parent FO. In SF, C18:1, C18:2, C18:3, C20:1, C20:5 (EPA), C22:1, C22:2 and C22:6 (DHA) increased to 9.84%, 24.35%, 61.09%, 32.10%, 39.96%, 56.81%, 39.02%, and 48.94% than parent FO. EPA (12.91 mg/100 g) and DHA (15.98 mg/100 g) contents were developed for the first time, and their suitability for developing *n‐3* enriched foods, especially infant and baby foods, should be studied comprehensively. To our knowledge, SF possesses the highest content of *n‐3* among all the dietary sources, *n‐3*, except ALA of plant origin (flaxseed, chia), which has far less bioavailability than EPA and DHA (Sanders 2000). Concentrations of C18:1 in FO, OF and SF were 25.72, 27.91 and 28.53 mg/100 g. This fatty acid is believed to have cardioprotective effects, and oils possessing reasonable C18:1 have superior oxidative stability than PUFA‐enriched oils (Anwar et al. 2007). For the production of a high oleic acid version of sunflower oil, winterization was employed, and oleic acid content increased from 14%–39.4% to 75%–90.7% (Ghazani and Marangoni 2013). Concentrations of saturated fatty acids in FO, OF, and SF were 30.19%, 15.55%, and 8.80%. The impact of winterization on the fatty acid profile of vegetable oils and fats has been extensively reported in the literature. Chia oil was cooled to −40°C to produce olein and stearin fractions. Both the fractions had considerably different fatty acid profiles from the native chia oil. Saturated fatty acids were intensified in stearin, while unsaturated fatty acids were intensified in olein fraction (Ullah et al. 2016). In the present study, the impact of long‐term ambient storage on fatty acid profile, FO, OF and SF was also studied; GC–MS analysis revealed that the storage phase of 90 days did not affect saturated fatty acids of FO, OF and SF; however, concentrations of unsaturated fatty acids significantly decreased in 3 months old samples of FO, OF and SF. Analysis of samples at the end of the months storage phase indicates that the loss of unsaturated fatty acids in FO, OF and SF was 6.87%, 7.32%, and 7.89%. In 3 months old FO, OF and SF, EPA contents were 6.42, 8.13 and 11.53 mg/100 g. In 3 months old FO, OF and SF, DHA contents were 7.62, 10.76 and 14.29 mg/100 g. In 3 months old FO, OF and SF, C22:1 contents were 0.17, 0.31 and 0.35 mg/100 g. In 3 months old FO, OF and SF, C22:2 contents were 0.17, 0.31 and 0.35 mg/100 g. The oleic acid content of *Moringa oleifera* oil was increased by winterization, the impact of 3 months of storage on the fatty acid profile was studied, and old samples had lower concentrations of unsaturated fatty acids than the initial values (Rahman et al. 2014).

**TABLE 2:** Fatty acid profile of FO, OF, and SF.

Fatty acid	FO		OF		SF	
	0‐day	90‐days	0‐day	90‐days	0‐day	90‐days
C14:0	4.91 ± 0.14^A^	4.85 ± 0.02^A^	3.61 ± 0.11^B^	3.55 ± 0.06^B^	1.15 ± 0.05^C^	1.12 ± 0.02^C^
C16:0	13.22 ± 0.23^A^	13.15 ± 0.01^A^	8.44 ± 0.51^B^	8.29 ± 0.39^B^	3.27 ± 0.19^C^	3.22 ± 0.02^C^
C16:1	9.43 ± 0.07^A^	9.32 ± 0.25^A^	7.19 ± 0.05^B^	7.05 ± 0.18^B^	3.83 ± 0.27^C^	3.75 ± 0.16^C^
C18:0	2.63 ± 0.04^A^	2.58 ± 0.07^A^	1.92 ± 0.03^B^	1.86 ± 0.08^B^	0.75 ± 0.02^C^	0.71 ± 0.01^C^
C18:1	25.72 ± 0.84^A^	25.44 ± 0.93^A^	27.91 ± 0.95^B^	26.51 ± 0.71^C^	28.53 ± 0.77^A^	27.16 ± 1.12^B^
C18:2	2.64 ± 0.09^E^	2.35 ± 0.11^F^	3.28 ± 0.22^B^	2.98 ± 0.06^D^	3.49 ± 0.09^A^	3.10 ± 0.24^C^
C18:3	1.21 ± 0.17^D^	1.12 ± 0.04^E^	2.64 ± 0.06^B^	2.41 ± 0.10^C^	3.11 ± 0.08^A^	2.62 ± 0.04^B^
C20:1	13.88 ± 0.39^E^	12.51 ± 0.46^F^	16.89 ± 0.54^C^	15.77 ± 0.79^D^	20.46 ± 0.64^A^	19.36 ± 0.57^B^
C20:5 (EPA)	7.75 ± 0.10^E^	6.42 ± 0.64^F^	9.26 ± 0.31^C^	8.13 ± 0.55^D^	12.91 ± 0.79^A^	11.53 ± 0.34^B^
C22:1	0.92 ± 0.02^E^	0.74 ± 0.05^F^	1.56 ± 0.03^C^	1.29 ± 0.01^D^	2.13 ± 0.52^A^	1.74 ± 0.03^B^
C22:2	0.25 ± 0.01^E^	0.17 ± 0.01^F^	0.37 ± 0.07^C^	0.31 ± 0.05^D^	0.41 ± 0.06^A^	0.35 ± 0.02^B^
C22:6 (DHA)	8.16 ± 0.65^E^	7.62 ± 0.28^F^	11.64 ± 0.88^C^	10.76 ± 0.34^D^	15.98 ± 0.48^A^	14.29 ± 0.29^B^

*Note:* In rows of individual fatty acids, when means bear different letter, it means these are statistically different (*p* < 0.05).

### 3.3 Total Phenolic Contents (TPC)

**FIGURE 1:**
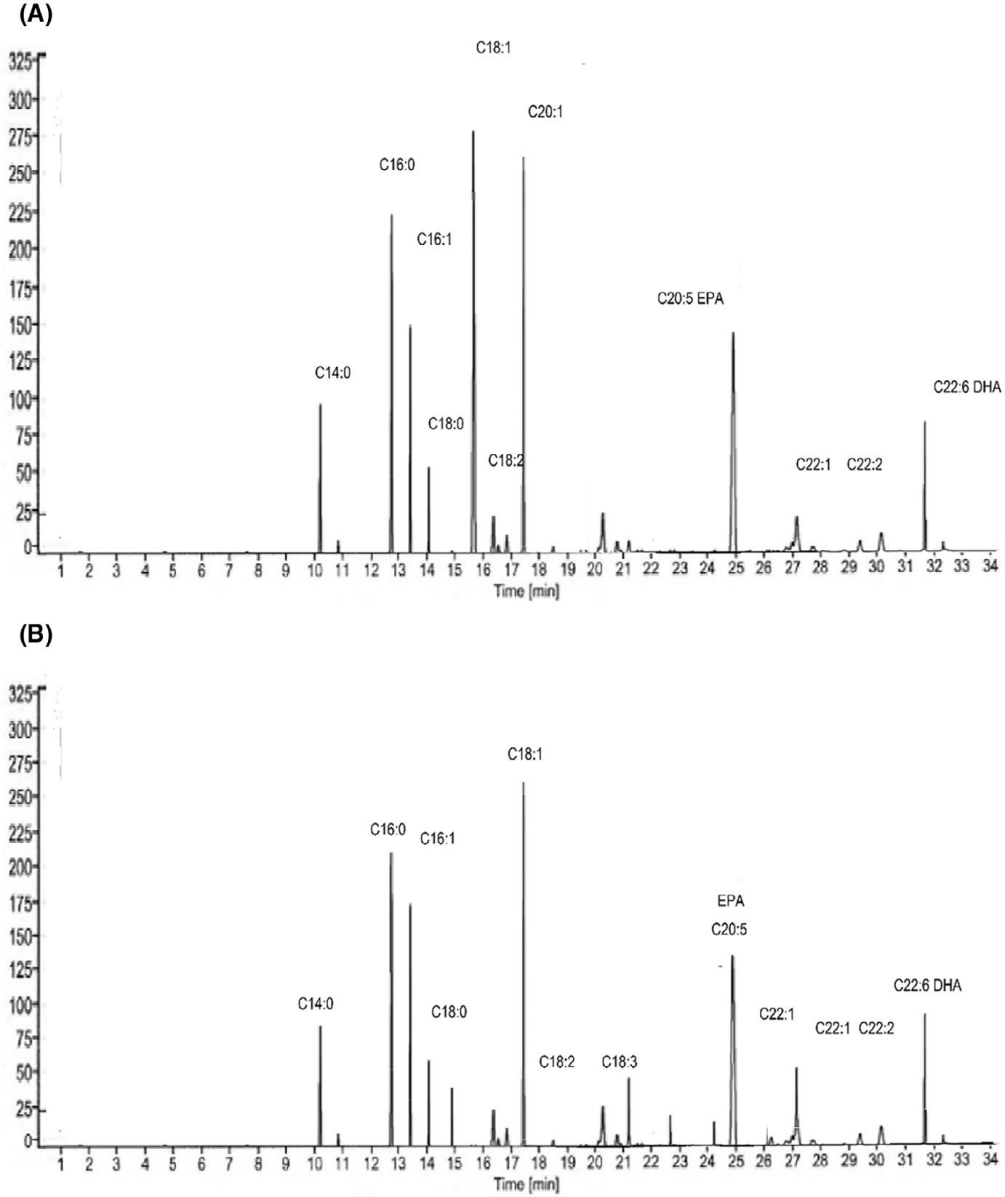
Fatty acid profile of FO at 0 day (A) and 90 days (B).

**FIGURE 2:**
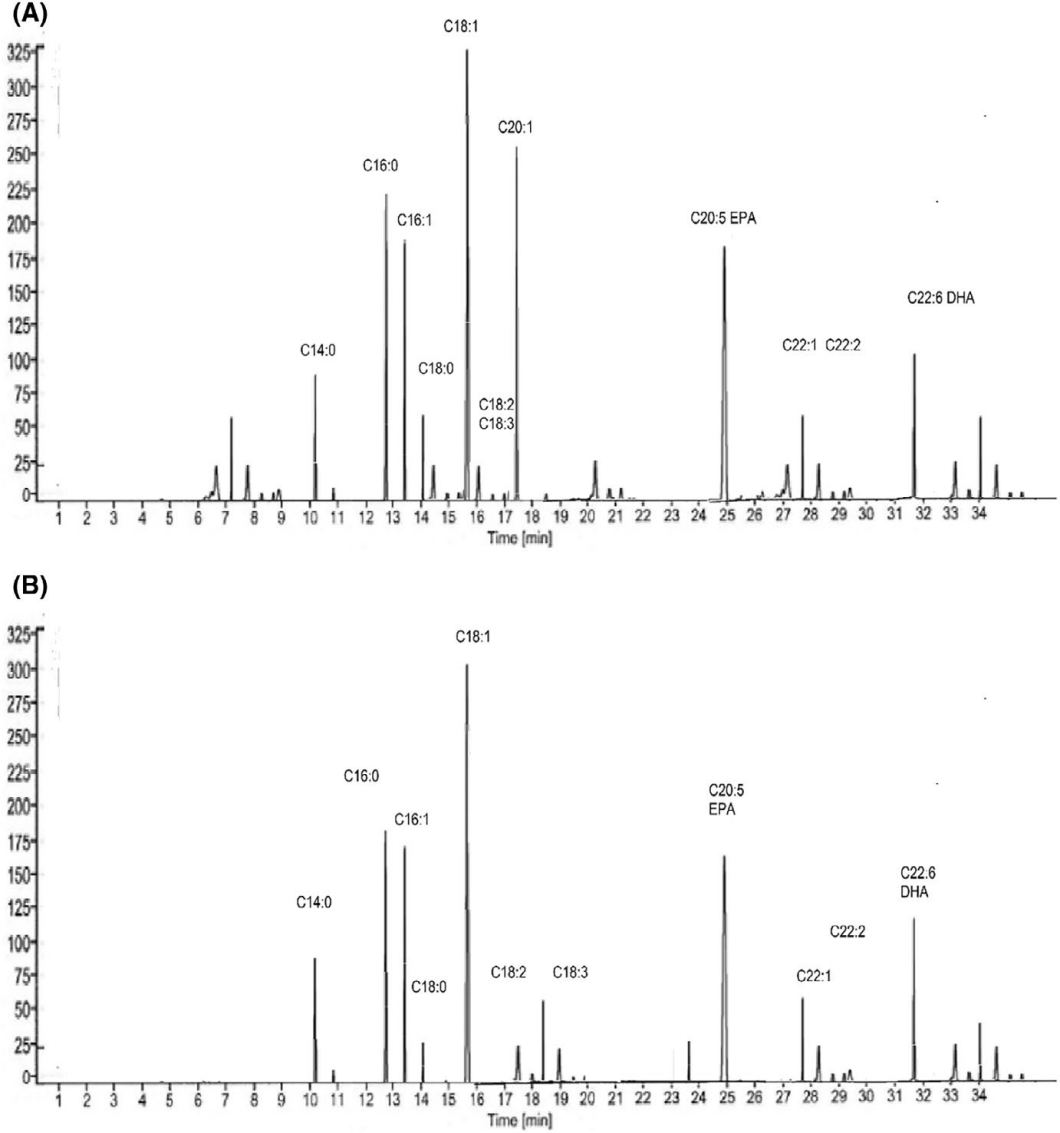
Fatty acid profile of olein fraction at 0 day (A) and 90 days (B).

**FIGURE 3:**
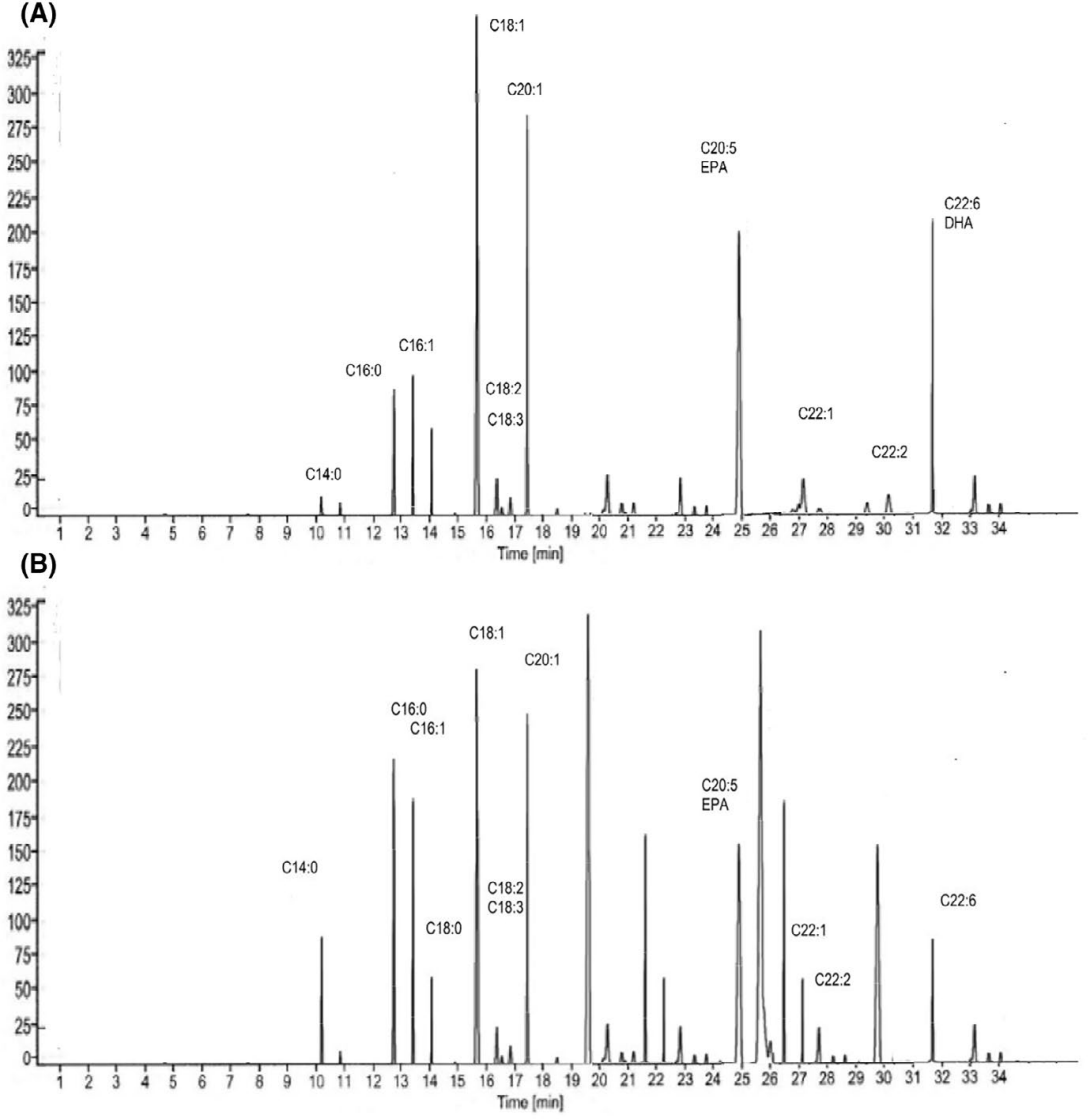
(A) Fatty acid profile of super olein fraction at 0 day. (B) Fatty acid profile of olein fraction at 90 days.

**FIGURE 4:**
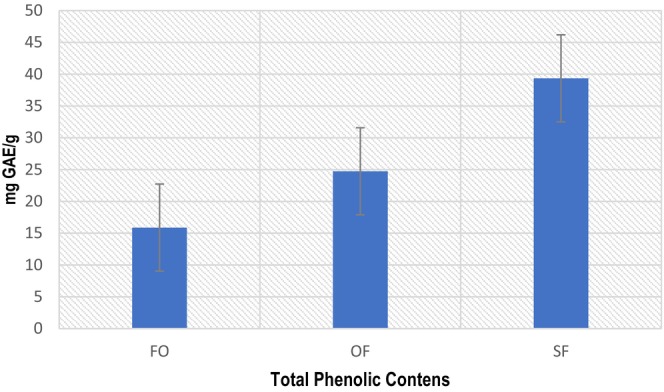
Total phenolic contents (TPC).

TPC estimation determines the quantity of phenolic compounds in the sample; phenolic compounds of plant origin have oxidation–reduction properties, letting them act as antioxidants. As *n*‐3 enriched oils are much more prone to lipid oxidation, naturally occurring phenolic compounds can stabilize *n‐3* fatty acids (De la Rosa et al. 2019). TPC of FO was 6.59 (mgGAE/mL), and winterization sequentially raised TPC in OF and SF due to the relationship of phenolic compounds with low melting point glycerides; thus, phenolic compounds were concentrated in OF and SF. The TPC contents of OF and SF were 12.67 and 19.72 (mgGAE/mL), respectively, represented in Figure 1A. Intensification of phenolic compounds in winterized oils is reported in the literature; single and double‐fractionated oleins of safflower oil had higher amounts of TPC than safflower oil (Khan et al. 2018). The TPC content of FO found in the present study is almost similar to those reported by (Rahim et al. 2023). The coefficient of correlation among TPC and TAC of FO, OF and SF were *R* ^2^ = 0.9414, 0.9711 and 0.9837, and the coefficient of correlations among TPC and DPPH of FO, OF and SF were *R* ^2^ = 0.9548, 0.989 and 0.9913, respectively. Several studies have concluded that phenolic compounds of oils can play a pivotal role in preventing metabolic ailments (Gutierrez‐Grijalva et al. 2017). Thermal processing affects The TPC of oils, and TPC decreased from 21.2 to 20 (mgGAE/100 g) in microencapsulation of propolis oil (Da Silva et al. 2013). In the current investigation, a non‐thermal method (winterization) was adopted to produce OF and SF; both the fractions had higher TPC than FO (Figures 2, 3, 4).

### 3.4 Lecithin

Lecithin is an emulsifier produced during the degumming of wet gums during oil extraction (Xie and Dunford 2017). It is a co‐product of the vegetable oil industry, extracted from crude oil through solvent extraction and degumming methods (Bueschelberger 2004). It is a mixture of different phospholipids PE, PC, PI, and PA; among them, phosphatidylcholine is the most common; these phospholipids are used in food, cosmetic and pharmaceutical industries in a wide variety (Joshi, Paratkar, and Thorat 2006). Other minor components like triglycerides, phenols, glycolipids, fatty acids and sterols are also present (Johnson Jr et al. 2020). The beneficial lipids present in lecithin make it an excellent food additive. These phospholipids derived from marine sources are essential in different body functions (Ramadan 2008). Phosphatidylcholine helps to increase the process of apoptosis and decrease cell proliferation and aging (Fukunaga, Hossain, and Takahashi 2008). The greater amount of phosphatidylcholine helps to release serum‐free choline in the body. Choline is not produced naturally in our body, although it is a precursor of Ach. This intake can be fulfilled by taking lecithin (Leblanc et al. 2003). Marine sources contain omega‐3 (*n‐3*) fatty acids, which greatly interest food industries (Alhajj et al. 2020). In oil–water emulsions, lecithin helps reduce surface tension and disrupt bulk phases into small droplets (Arrigo and Servi 2012). Lecithin is a universal emulsifier used in infant formula milk; confectionary items and baked goods include bread and chocolate coatings (List 2015). The lecithin (phospholipid) content of FO was 1.29%, winterization of FO at −50°C to produce olein, and the second stage of winterization of OF to produce SF had a major effect on lecithin content. Lecithin content of OF and SF was significantly less than FO. Lecithin contents of OF and SF were 0.57% and 0.19%, respectively. The reduction in lecithin content of OF and SF was due to its solidification at −50°C and −75°C employed for winterization; it was solidified along with high melting point glycerides, and most of the lecithin was removed during the separation of liquid and solid phases (Brevedan, Crapiste, and Carelli Albarracin 2000). At industrial scale, the removal of phospholipids from vegetable oils is employed to produce salad oils and oils that do not get hazy in the winter season and further improve the storage stability, prevent foam in frying operation, prevent darkness in the deodorization stage and increased the efficiency of bleaching earth in bleaching process (Ruiz‐Mendez, Aguirre‐Gonzalez, and Dobarganes 2011).

### 3.5 Sterols in FO, OF, and SF

GC–MS analysis of FO, OF and SF showed the existence of desmosterol, cholesterol, stigma sterol and sitosterol in considerably different amounts. All kinds of detected sterols increased with the progression of winterization. In OF, magnitudes of desmosterol, cholesterol, stigma sterol and sitosterol were 74.14, 15.37, 8.37 and 82.38 mg/100 g (Table 3). In SF, desmosterol, cholesterol, stigma sterol and sitosterol were 91.57, 22.51, 12.67 and 112.18 mg/100 g. FO oil had the lowest sterols content. In FO, desmosterol, cholesterol, stigma sterol, and sitosterol were 68.19, 9.42, 5.21 and 63.79 mg/100 g. The rise in desmosterol, cholesterol, stigma sterol, and sitosterol in OF and SF was due to their affiliation with glycerides having low melting points, which were intensified in OF and SF. Sterols are an essential part of unsaponifiable matter and constitute a minor part of oils with no major impact on their functionality and physical properties; however, they have strong oxidative stabilization properties (Jin et al. 2018). Palm oil was fractionated to produce OF, and SF and sterol content followed this pattern SF > OF > palm oil (Ok 2014). Concentrations of desmosterol, cholesterol, stigma sterol and sitosterol in FO were 6.84, 32.21, 4.19 and 38.32 mg/100 g (Zhang et al. 2019). Concentrations of sterols in FO observed in this study differed slightly from vegetable oils such as palm oil and soybean oil (Hamm, Hamilton, and Calliauw 2013). Chia oil was subjected to winterization to produce olein fractions, and the sterol content of the olein version was considerably higher than that of mother chia oil (Ullah et al. 2018). Three tailored versions of milk fat were produced by winterization at 25°C, 15°C, and 10°C. All these fractions showed higher sterols content than the starting material (Nadeem, Situ, and Abdullah 2015).

**TABLE 3:** Sterols (mg/100 g) of olein, super olein and FO.

Sterol	FO	OF	SF
Desmosterol	68.19 ± 0.13^C^	74.14 ± 0.09^B^	91.57 ± 0.27^A^
Cholesterol	9.42 ± 0.25^C^	15.37 ± 0.05^B^	22.51 ± 0.06^A^
Stigmasterol	5.21 ± 0.04^C^	8.37 ± 0.02^B^	12.67 ± 0.09^A^
Sitosterol	63.79 ± 0.49^C^	82.38 ± 0.16^B^	112.18 ± 0.20^A^

*Note:* In rows of individual parameters, when means bear different letter, it means these are statistically different (*p* < 0.05).

### 3.6 Antioxidant Capacity

The impact of single‐ and two‐stage winterizations on the antioxidant characteristics of OF and SF production is shown in Table 4. TAC and DPPH of OF and SF depended upon the presence and intensification of phenolic compounds and other antioxidant substances in these fractions. Magnitudes of phenolic compounds, vitamins A, E and other antioxidative compounds were significantly higher in SF than OF and FO, respectively. The sequential rise of the compounds in present study, SF and OF increased their TAC and DPPH capacities. In fresh form, FO, OF and SF, TAC were 49.32%, 64.27%, and 85.47% (*p* < 0.05). In fresh form, the DPPH of FO, OF and SF was 32.14%, 39.87%, and 46.41%. TAC and DPPH of FO, OF and SF remained non‐significant till 45 days, then TAC and DPPH dropped, and 90 days samples showed a remarkable decline in their values (*p* < 0.05). In 3 months, in old samples, the TAC of FO, OF and SF was 42.29%, 52.16%, and 76.26%, while DPPH of 3 months FO, OF, and SF was 27.63%, 32.54%, and 40.11%. To estimate the efficiency of antioxidants and keep the quality of formulated foods and their ingredients, TAC measurement allows the Research and Development teams to make clear‐cut decisions regarding the working of antioxidants and anticipated storage oxidative stability. The greater the TAC value, the better the oxidative stability of foods (Sies 2007). Suppose a bioactive compound having a higher TAC value is added to a relevant food. In that case, it also increases the TAC of supplemented foods. Ice cream was produced from the olein fractions of chia oil, and compared with standard ice cream produced from milk fat, the former had higher antioxidant capacity due to the intensification of antioxidative compounds (Ullah et al. 2018). Another reason for the increase of TAC and DPPH of OF and SF compared to FO is the intensification of vitamins A and E in these fractions. The antioxidant activity of both these vitamins is scientifically established (Khan et al. 2019). DPPH of pure and FO and chia oil blends were more than 30% and 90% (Rahim et al. 2023). To determine the efficiency of antioxidant substances in a food matrix, the DPPH test is preferentially performed in several food service establishments due to its accuracy, reliability, and acceptability (Anwar et al. 2010). To produce *trans*‐free, *n‐3* enriched margarine, chia oil was blended with palm oil, palm olein and palm kernel oil. Experimental margarine had higher TAC than margarine produced without adding chia oil (Nadeem et al. 2017). The impact of 3 months of storage on TAC and DPPH of OF and SF of date seed oil produced by winterization, 3‐month‐old samples of OF and SF showed significantly lower values of TAC and DPPH than the starting values (Hussain et al. 2022). Antioxidant studies of rapeseed oil were conducted through ORAC and FRAP assays, and it was found that the values of extracted rapeseed oil were higher than the AC of pressed rapeseed oils (Szydlowska‐Czerniak et al. 2008). Virgin coconut oil extracted through fermentation shows better antioxidant capacity than refined oil (Marina et al. 2009). Due to saturated fatty acids, palm olein shows higher thermo oxidative stability than soybean and canola oil (Memon et al. 2022).

**TABLE 4:** Antioxidant capacity of FO, OF, and SF.

Treatment	Days in storage	TAC%	DPPH%
FO	0	49.32 ± 0.32^E^	32.14 ± 0.22^E^
	45	48.82 ± 0.21^E^	31.97 ± 0.16^E^
	90	42.29 ± 1.13^F^	27.63 ± 0.07^F^
OF	0	64.27 ± 0.79^C^	39.87 ± 0.67^C^
	45	63.59 ± 0.53^C^	38.66 ± 0.49^C^
	90	52.16 ± 0.89^D^	32.54 ± 0.26^D^
SF	0	85.43 ± 0.26^A^	46.41 ± 0.38^A^
	45	84.22 ± 0.61^A^	45.72 ± 0.54^A^
	90	76.26 ± 0.39^B^	40.11 ± 0.03^B^

*Note:* In rows of individual parameters, when means bear different letter, it means these are statistically different (*p* < 0.05).

### 3.7 Vitamin A and E

Fat‐soluble vitamins perform several physiological functions in the human body. Vitamin A (retinol) regulates the expression of genes, photoreception, growth of teeth, and development of bones. Vitamin E is one of the most potent antioxidant compounds in the body that prevents the oxidation of vitamin A, essential fatty acids, anti‐aging effects and lipoproteins in the membrane. American Heart Association directs people to consume at least two servings of fish per week; however, in several countries, the recommended amount is not consumed due to several financial, cultural, availability, and sustainability issues. Communities taking inadequate fish cannot get beneficial vitamins A, E, *n*‐3 and numerous bioactive compounds (Ribarova 2007; FAO/WHO 2013) The vitamin A content of common carp and rainbow trout was 30 IU and 280 IU (Atanasov et al. 2009). Vitamin E content in trout and mullet fillets were 1112 and 1274 μg/100 g (Ahmadnia et al. 2008). FO was winterized with and without solvents to concentrate the *n‐3*. Liquid fraction had higher *n‐3*. However, the impact of winterization on FO's vitamin A and E contents is not previously reported in the literature (Tengku‐Rozaina and Birch 2013). Vitamins A and E belong to the unsaponifiable fraction. As a result of sequential winterization involved in the production of OF and SF, unsaponifiable matter increased as the winterization proceeded. Unsaponifiable matter in FO, OF and SF was 1.77%, 1.92% and 2.16%. Food industries and researchers are facing continuous pressure to increase food's functional value and alter the composition of existing foods so that these become functional foods. In the present study, an effort was made to increase the functional value of FO to convert it to a functional ingredient with increased amounts of beneficial *n‐3*, vitamins A, E, and so forth. As the concentration of vitamin A remarkably increased in SF, it can be used as a substrate for producing vitamin A concentrate instead of FO. The saponification of triglycerides produces a concentrate of vitamin A extracted from unsaponifiable matter. This method led to a massive loss of neutral oil, which increase the waste production cost of vitamin A concentrate and is not recommended in the current food insecurity situation. The yield of SF was 23%. Using SF as a substrate for vitamin A production at the commercial level can prevent the loss of about 77% of precious FO for food‐insecure nations. Winterization significantly raised vitamins A and E in OF and SF. Vitamin A content in FO, OF and SF (0‐day) were 46.28, 67.94, and 116.48 IU, vitamin E content in FO, OF and SF (0‐day) were 1238.95, 1897.65 and 2375.11 mg/100 g (Table 5). Storage of FO, OF and SF under ambient conditions did not show any reducing effect on vitamin A and E till half of the storage; however, 90‐day‐old samples of FO, OF and SF had lower vitamin A and E than the preliminary values (*p* < 0.05). In 3‐month‐old samples of FO, OF, and SF, vitamin A was 40.29, 62.49, and 116.48 IU. In 3‐month‐old samples of FO, OF, and SF, vitamin E was 1088.91, 13.54.82, and 19.36.95 (mg/100 g). Olein and stearin fractions of palm oil were separated to know the concentrations of bioactive compounds. α and β‐carotene in olein fraction were 232 and 213 mg/kg, α and β‐carotene in stearin fraction were 347 and 299 mg/kg, and α‐tocopherol in olein and stearin fractions was 136 and 90 mg/kg (Guedes et al. 2023). Red palm oil was winterized to produce palm olein, vitamin E and carotenoids were studied in red palm oil and palm olein, and vitamin E in red palm oil and palm olein was 171 and 218 mg/100 g, respectively. β‐carotene in red palm oil and palm olein was 542 and 874 mg/100 g (Top et al. 2011). Oil extracted from *Lates niloticus* has the highest vitamin A content of 3.90 ± 0.02 to 5.9/100 mg (Ogwok, Muyonga, and Sserunjogi 2008). The α‐tocopherol and carotenes in Tecolote ray range between 2.9–17.4 mg/100 g and 3.0–15.2 mg/100 g (Navarro‐Garcıa et al. 2004). Winterization of Nile perch viscera oil reveals no significant difference among all three fractions (melting point, smoking point and density) of vitamin E, whereas significant differences were observed in vitamin A content (Okoth, Imungi, and Aloo 2015). Victoria Nile Perch oil was winterized to obtain LMP and HMP fractions. No significant difference was observed in vitamin E or A (Aloo 2014).

**TABLE 5:** Vitamin A and E of FO, OF, and SF.

Treatment	Days in storage	Vitamin A (IU)	Vitamin E (mg/100 g)
FO	0	46.28^E^	1238.95^E^
	45	45.34^E^	1263.79^E^
	90	40.29^F^	1088.91^F^
OF	0	67.94^C^	1897.65^C^
	45	66.54^C^	1877.62^C^
	90	62.49^D^	1354.82^D^
SF	0	116.48^A^	2375.11^A^
	45	114.79^A^	2344.12^A^
	90	108.36^B^	1936.95^B^

*Note:* In rows of individual parameters, when means bear different letter, it means these are statistically different (*p* < 0.05).

### 3.8 Lipid Oxidation

About 98% of FO comprises TAGs with three fatty acids attached to the glycerol backbone. Lipases, water, metal ions, elevated storage temperature, etc., may cause fatty acid separation from glycerol molecules. It is referred to as hydrolysis and rancidity caused by excessive amounts of free fatty acids, known as hydrolytic rancidity (O'Brien 2008). To a certain limit, FFA does not generate bad smells and deteriorate the sensory properties of foods. The threshold level of FFA per the European Union guidelines is 0.2% max. When the FFA level of foods exceeds this threshold level, they start giving off a rancid smell, and foods become unacceptable. Therefore, during the developmental phase of functional foods and their multiplication at the industrial level, the concentration of FFA is seriously considered; ingredients/raw materials with lower amounts of FFA are used to formulate functional foods (Zhang et al. 2006). At the industrial level, FFA from FO and vegetable oils is decreased by Alkali refining/physical refining. This is only possible in edible oil refineries, and this job cannot be done in most food processing industries using edible oils in their formulations. Therefore, they rely on the FFA of incoming raw materials. In addition to inducing bad smells in foods, FFA can also trigger the oxidative breakdown in FO, destroying the sensory profile of foods (Imran et al. 2022). According to food and Drug Administration (FDA), 2‐g *n* *‐* *3* fatty acids should be taken on regular basis. FO is considered a rich source of *n* *‐* *3* however most of the unprocessed FO is unpleasant due to the fishy flavor, in addition it cannot be used in processed foods. In this regard, refining, deodorization and bleaching are required to produce food grade FO. This leads to production of polymers and bioactive compounds. Winterized version of flaxseed oil, chia seed oil safflower oil, palm oil, and date seed has been documented in literature but FO winterized form has been studied in limited way. Therefore, to make the fortification strategies sustainable, alternate sources like FO should be investigated for possible food applications. It is scientifically established that FFA has no link with PUFA (extent of unsaturation). Hydrolysis can happen in any TAGs with any specificity of unsaturation (Azeem, Nadeem, and Ahmad 2015). In this study, the same trend was found: first and second winterization for the production of OF and SF did not cause any variation in FFA content, although the degree of unsaturation was in the order of FO > OF > SF. Fat was successively fractionated thrice to produce three different kinds of fractions. Each fraction had more unsaturated fatty acids than the previous fraction, with no difference in FFA content (Nadeem et al. 2014). FFA of FO, OF and SF were 0.13%, 0.12%, and 0.14% (*p* > 0.05) and remained non‐significant up to the half duration of storage. FFA of all variants was considerably higher than initial values with no mutual difference. The rise in FFA of FO, OF and SF was an age and storage‐related phenomenon without connection with double and triple bonds. FFA content of 3 months stored OF and SF were still in the range of the granted limit of the EU. As the number of fryings increased, the content of FFA palm olein increased in the oil samples (Nor et al. 2008). During deep frying, the degradation of free fatty acids occurs. Because of this, the content of FFA rises in the product. These products are not accepted commercially due to their off‐flavor (Maskan and Horuz 2017). In most deep frying conditions, the FFA produced during hydrolysis is too small to affect the overall palatability of food (Abdulkarim et al. 2007). The FFA content of Nile perch visceral oil generally increases with acid concentration and heating (Aloo 2014). End products of auto‐oxidation are aldehydes, ketones, alcohols, and so forth. Lipid oxidation products have potent detrimental health impacts. Nutritionists have warned consumers that oxidized lipids can be more dangerous than partially hydrogenated fats and excess saturated fatty acids (Arif et al. 2016). The EU requires strict compliance with POV (10 MeqO_2_/Kg) because of the matter's sensitivity. The AV of freshly deodorized oils should preferably be less than 10 (Shahidi 2005). By measuring POV and AV, the production of primary, secondary and tertiary oxidation products can be quantified (Chatha et al. 2011). With the advancement in analytical chemistry, accelerated methods of lipid oxidation measurement, such as the induction period by Rancimat, have been developed. This method is highly reliable and recommended by the American Oil Chemists Society; POV is still the most reliable method for measuring lipid oxidation due to its convenience, high reliability and reproducibility (Nadeem et al. 2013). In this study, the winterization of FO did not impact the POV and AV of OF and SF. In freshly produced FO, OF and SF (0‐day), POV was 0.22, 0.24 and 0.25 (MeqO_2_/Kg). In freshly produced FO, OF and SF (0‐day), AV was 6.17, 5.98 and 6.22. POV and AV remained insignificant until half of the storage phase (*p* > 0.05). After the completion of the storage phase, POV and AV were significantly higher than the values observed at 0 and 45 days of storage, and POV of 90 days old FO, OF and SF was 0.50, 0.61 and 0.75 (MeqO_2_/Kg). AV of 90 days old FO, OF and SF was 9.33, 11.26 and 13.47 (Table 6). PUFA are considered highly sensitive to lipid oxidation. In the current investigation, POV did not shoot up during 90 days of ambient storage and was much less than the granted limit of the EU. The slow rise of POV in OF and SF can be connected to the intensification of phenolic compounds, vitamin E, A, and sterols. All these compounds are believed to have an oxidative stabilization effect. The recommended AV of good quality FO should be < 20 (Hamilton et al. 1998). The POV of carp visceral oil was found to be 1.78 ± 0.01 (Crexi et al. 2010). The lowest POV of sardine oil observed after winterization was at 4°C, whereas the maximum POV was at 7°C (Suseno, Sintoko, and Fitriana 2017). The deodorized oil after the refining of Kilka fish and after winterization caused no effect on POV (Motalebi Moghanjoghi et al. 2015).

**TABLE 6:** Lipid oxidation of single and double fractionated olein of safflower oil.

Treatment	Days of storage	FFA%	POV (MeqO_2_/kg)	Anisidine value
FO	0	0.13 ± 0.02^B^	0.22 ± 0.02^D^	6.17 ± 0.05^D^
	45	0.14 ± 0.01^B^	0.26 ± 0.01^D^	6.44 ± 0.07^D^
	90	0.17 ± 0.01^A^	0.50 ± 0.01^C^	9.33 ± 0.02^C^
OF	0	0.12 ± 0.01^B^	0.24 ± 0.03^D^	5.98 ± 0.09^D^
	45	0.13 ± 0.03^B^	0.27 ± 0.04^D^	6.64 ± 0.06^D^
	90	0.16 ± 0.01^A^	0.61 ± 0.02^B^	11.26 ± 0.12^B^
SF	0	0.14 ± 0.02^B^	0.25 ± 0.04^D^	6.22 ± 0.16^D^
	45	0.15 ± 0.01^B^	0.26 ± 0.01^D^	6.73 ± 0.09^D^
	90	0.18 ± 0.01^A^	0.75 ± 0.03^A^	13.47 ± 0.16^A^

### 3.9 Sensory Evaluation

In a specialized sensory evaluation laboratory, a panel comprising 10 trained judges could not find any significant difference in color and smell of FO, OF and SF when tested at 0, 45, and 90 days. None of the panelists reported oxidized flavor in any sample at all evaluation stages. Color of SO, OF and DFO was 7.6, 7.7 and 7.7 and smell score FO, OF, and SF were 7.8, 7.7, and 7.6, respectively. From the results of color and smell, it can be perceived that FO, OF, and SF may not affect the color and smell score of the foods. The smell of FO, OF, and SF was bland and resembled commercially deodorized oils. Sensory evaluation of 90‐day‐old samples showed that the FO, OF, and SF color and smell scores remained unchanged (Table 7).

**TABLE 7:** Sensory characteristics of FO, OF, and SF.

Treatment	Days of storage	Color	Smell
FO	0	7.6 ± 0.04	7.8 ± 0.21
	45	7.5 ± 0.13	7.7 ± 0.16
	90	7.5 ± 0.14	7.6 ± 0.05
OF	0	7.7 ± 0.15	7.7 ± 0.13
	45	7.6 ± 0.18	7.6 ± 0.19
	90	7.5 ± 0.10	7.7 ± 0.09
SF	0	7.7 ± 0.16	7.6 ± 0.25
	45	7.5 ± 0.08	7.6 ± 0.17
	90	7.6 ± 0.17	7.8 ± 0.07

*Note:* If in a single column of this table, means have a dissimilar letter, it shows significant difference (*p* < 0.05).

## 4 Conclusion

In the present investigation, the super olein fraction of fish oil (FO) was first time produced by winterization. EPA and DHA contents of super olein fractions were 39.7% and 48.9% higher than parent FO, respectively. In the olein fraction, EPA and DHA were 16.3% and 29.8% more than substrate FO, respectively. After 90 days of ambient storage, the sensory properties of free fatty acids, peroxide value, and super olein fractions were similar to those of FO. Omega‐3 (*n‐3*) fatty acids can be successfully increased in the super olein fraction of FO by winterization.

## Author Contributions

Aiza Khaliq and Muhammad Nadeem made significant contributions to conducting the research and played a primary role in preparing this article. Muhammad Hafeez‐ur‐Rehman and Farzana Abbas contributed to the write‐up and provided all the facilities to conduct research under her supervision. Fahad Al‐Asmari and Mohamed Fawzy Ramadan played a crucial role in interpreting the results and augmenting the manuscript. Fahad Al‐Asmari, Eliasse Zongo, Muhammad Nadeem, and Muhammad Abdul Rahim contributed to the statistical analysis and helped edit and review the manuscript.

## Conflicts of Interest

The authors declare no conflicts of interest.

## Declaration

I/we hereby declare that this research work has not been published elsewhere, nor is it under consideration for any other publication.

## Data Availability

Data is contained within the article.
